# Curdione Induces Antiproliferation Effect on Human Uterine Leiomyosarcoma *via* Targeting IDO1

**DOI:** 10.3389/fonc.2021.637024

**Published:** 2021-02-26

**Authors:** Chao Wei, Donghua Li, Yu Liu, Wenna Wang, Tiantian Qiu

**Affiliations:** School of Traditional Chinese Medicine, Capital Medical University, Beijing, China

**Keywords:** curdione, uterine leiomyosarcoma, apoptosis, autophagy, Indoleamine-2, 3-dioxygenase-1

## Abstract

**Objectives:**

Curdione is one of the active ingredients of a traditional Chinese herbal medicine-*Curcuma zedoary* and established anti-tumor effects. Uterine leiomyosarcoma (uLMS) is a rare gynecological malignancy, with no standard therapeutic regimen at present. The aim of this study was to explore the potential anti-tumor impact of curdione in uLMS and elucidate the underlying mechanisms.

**Methods:**

*In vitro* functional assays were performed in the SK-UT-1 and SK-LMS-1 cell lines. The *in vivo* model of uLMS was established by subcutaneously injecting SK-UT-1 cells, and the tumor-bearing mice were intraperitoneally injected with curdione. Tumor weight and volume were measured at specific time points. The biosafety was evaluated by monitoring changes of body weight and the histopathology in the liver and kidney. The expression levels of relevant proteins were analyzed by western blotting and immunohistochemistry.

**Results:**

Curdione decreased the viability and proliferation of uLMS cells in a concentration and time-dependent manner. In addition, the curdione-treated cells exhibited significantly higher rates of apoptosis and autophagic death. Curdione also decreased the tumor weight and volume in the SK-UT-1 xenograft model compared to the untreated control without affecting the body bodyweight or pathological injury of liver and kidney tissues. At the molecular level, the anti-tumor effects of curdione were mediated by indoleamine-2, 3-dioxygenase-1 (IDO1).

**Conclusion:**

Curdione exhibited an anti-uLMS effect *in vitro* and *in vivo*; the underlying mechanism involved in IDO1 mediate apoptosis, autophagy, and G2/M phase arrest.

## Introduction

Uterine leiomyosarcoma (uLMS) is a rare and aggressive gynecological malignancy, which is characterized by high recurrence rate, low mortality rate, distant metastases, and poor prognosis (1, 2). It is the most common subtype of uterine sarcoma, and clinical manifestations are abnormal uterine bleeding, palpable pelvic mass, and lower abdominal pain. Since these symptoms mimic uterine leiomyoma, uLMS is often misdiagnosed (3–5).

In addition, no standardized therapeutic regimen has been available for uLMS due to its rare occurrence and rapid progression (6, 7), effective and standardized therapy is urgently needed.

Herbal medicine exhibits broad-spectrum antibacterial, anti-inflammatory, and anticancer activity with tolerable toxicity, which plays a pivotal role in preventing and treating disease. Currently, chemotherapy is one of the main strategies against cancer; herbal medicine gained considerable interest in recent years as an alternative to chemotherapy drugs. For instance, *Curcuma zedoary* has been reported with an anti-tumor effect (8–11), and its bioactive compounds of the essential oils including *β*-elemene, curcumol, curcumin, curdione, furanodiene, furanodienone, and germacrone exhibit anti-thromboric (12, 13), anti-inflammatory, (14) antibacterial (15), neuroprotective properties (16), cardio-protective (17), and anti-tumor effects (18). The therapeutic effects of curdione have been reported in breast cancer (19), while the potential anti-tumor effect in uLMS is still unclear.

Evading death and uncontrolled proliferation are hallmarks of the tumor. The death forms of tumor cells include but not limited to apoptosis, autophagy, and necrosis. Apoptosis is a form of programmed cell death (20), and the most common type of tumor cell death induced by natural plant-derived compounds in tumor cells (21). It can initiate cascade of caspases reaction through both intrinsic (mitochondrial) or extrinsic (death receptor) pathways, and eventually cell death (22). The intrinsic apoptosis pathway is commonly triggered by hypoxia, chemotherapy, and radiation. Phytochemicals have multi-target effects on tumor; in addition to activating apoptosis, they can also induce autophagy through epigenetic mechanisms (23). It is a self-catabolic process that is activated in response to stress and maintains cellular homeostasis by degrading unnecessary substances such as misfolded proteins or damaged organelles. In addition, autophagy is the second-most common form of tumor cell death induced by phytochemicals (24). Nevertheless, autophagy acts as a double-edged sword in tumor cells by fulfilling their nutrient and energy requirements in the hypoxic or energy shortage environment on one hand and inducing cell death after sustained hyper-activation on the other hand (25). Cell death is a complex and sophisticated process; typically, it may not be limited to a single form but involves multiple forms (24).

Indoleamine-2, 3-dioxygenase-1 (IDO1) is an immune checkpoint and a key rate-limiting enzyme that breaks down tryptophan into kynurenine, which plays an important regulatory effect in tumor immunity (26). It is overexpressed in many tumor types, but not all. Liu et al. (27) reported significantly higher expression of IDO1 in uterine carcinosarcoma and uterine corpus endometrial carcinoma tissues compared to the paired normal tissues. In addition, IDO1 overexpression is related to increased tumor progression and poor prognosis of tumors, which makes it a promising therapeutic strategy for malignant tumors (28). Generally, the effect of IDO1 on tumors depends on its mediate immunoregulatory effect; however, Thaker (29) found that IDO1 directly mediates the proliferation and progression of colon cancer independent of its immunoregulatory effects. In our preliminary experiments as well, we detected high IDO1 expression in uLMS cell lines. We speculated whether the effect of curdione on uLMS is through targeting IDO1. If so, how does IDO1 mediate the suppression effect of curdione on uLMS?

Thus, the goal of this study was to analyze the effect of curdione on uLMS cells and the possible mechanistic role of IDO1. In this work, curdione inhibited the growth of SK-UT-1 and SK-LMS-1 cells by inducing cell cycle arrest at the G2/M phase, as well as apoptosis and autophagic death. Intrinsic apoptosis induced by curdione was demonstrated by the increased levels of cleaved caspases 3, 6, and 9 but that of pro-and cleaved-caspase 8. *In vivo*, curdione suppressed the tumor growth in the SK-UT-1 xenograft model without adverse effects. In addition, it significantly down-regulated IDO1 expression in a dose and time-dependent manner. Pharmacological inhibition of IDO1 with epacadostat or its siRNA-mediated knockdown restored the viability of cells following curdione treatment. Likewise, co-treatment with epacadostat and curdione attenuated the curdione-induced apoptosis, autophagy, and G2/M phase arrest. This demonstrated that the suppressive effect of curdione on uLMS *via* targeting IDO1.

## Material and Methods

### Reagents and Materials

Curdione was purchased from Solarbio Science&Technology Co. Ltd. (Beijing, China). Modified Eagle’s medium (MEM), Dulbecco’s modified Eagle’s medium (DMEM), fetal bovine serum (FBS), Non-Essential Amino Acids (NEAA), Pymvate Sodium (NaP), and penicillin–streptomycin (PS) were purchased from Gibco (Waltham, MA, USA). The IDO1 inhibitor epacadostat was purchased from Selleck (S7910, Texas, USA) and autophagy inhibitor 3- Methyladenine was purchased from Selleck (S2767, Texas, USA), CCk8 was purchased from Dojindo (CK04, Kumamoto, Japan), Beyo Click™ Edu-594 Cell Proliferation Kit was purchased from Beyotime (C0078S, Shanghai, China), and Annexin V-fluorescein isothiocyanate (FITC) cell apoptosis kit was purchased from Invitrogen (V13241, New York, California, USA).

### Cell Culture

SK-UT-1 and SK-LMS-1 cells were obtained from American Type Culture Collection (Manassas, VA, USA). The SK-UT-1 cells were cultured in MEM, supplemented with 10% FBS, 1% NEAA, 1% NaP, and 1% penicillin–streptomycin. The SK-LMS-1 cells were grown in DMEM supplemented with 10% FBS and 1% penicillin–streptomycin. Both cell lines were incubated in a humidified atmosphere with 5% CO_2_ at 37°C.

### Cell Viability Assay

The viability of the suitably treated cells was detected by the CCK-8 kit. Briefly, the SK-UT-1 and SK-LMS-1 cells were starved with serum-free and then incubated with gradient concentrations of curdione for 24 h, or with 100 μM curdione for 24, 48, and 72 h. In another experiment, the serum-starved cells were pre-treated with 3-MA or eapacadostat for 2 h, and then with curdione or not for 24 h. 10μl CCK8 solution was added into each well, the cells were incubated for 2 h at 37°C. The optical density (OD) at 450 nm was measured at a microplate spectrophotometer (BioTek, USA).

### Immunofluorescence

EdU assay was performed using Beyo ClickTM Edu-594 Cell Proliferation Kit (Beyotime, Shanghai, China), according to the manufacturer’s instructions. Briefly, curdione-treated cells were incubated with EdU, fixed with 4% paraformaldehyde, and then permeabilized with 0.3% Triton X-100. Following incubation with click additive solution, the nucleus was counterstained with Hoechst33342 for 15 min. To detect Ki67 expression levels, the cells were incubated with the anti-Ki67 primary antibody (ab15580, Cambridge, UK) and then with fluorophore-conjugated secondary antibody for 1 h, followed by counterstaining with 4,6-diamino-2-phenyl indole (DAPI, Genview, Florida, USA) for 10 min. For the TUNEL assay, the cells were stained with the reagents provided with the TUNEL kit (KGA7072, KeyGen, Nanjing, China) according to the manufacturer’s instructions. The stained cells were observed under laser confocal microscope (Leica DM60008, Kyoto, Japan) at a magnification of ×40, and the number of EdU, Ki67, and TUNEL positive cells from six random fields was used for quantitative analysis by Image J software.

### Flow Cytometry Analysis

The serum-starved cells were exposed to curdione for 24 h and then were harvested and co-stained with Annexin V-FITC & PI cell apoptosis kit according to the manufacturer’s instruction. In addition, the cell cycle distribution was analyzed using the Cell Cycle and Apoptosis Analysis Kit (C1052, Beyotime, Shanghai, China) according to the manufacturer’s instructions. Briefly, the cells were fixed with 70% cold ethanol for 24 h and then incubated with 5 µl PI staining solution and 45 µl RNase water. Subsequently, all stained samples were analyzed by flow cytometry (BD Biosciences, CA, USA).

### Transient Transfection

IDO1-specific siRNA (5′-GGATGTTCATTGCTAAACA-3′) was designed and synthesized by RiboBio Co. Ltd. (C10511-05, Beijing, China). The SK-UT-1 and SK-LMS-1 cells were transfected according to the manufacturer’s instructions. The stably transfected cells were confirmed by analyzing the IDO1 protein and mRNA expression levels.

### Western Blotting

Total protein was extracted from tumor tissues and uLMS cells and quantified by the BCA Protein Assay kit (Beyotime, Shanghai, China). Equal amounts of proteins (30 μg) per sample were separated by SDS-PAGE (Solarbio, Beijing, China) and then transferred to polyvinylidene fluoride microporous membranes (PVDF) (XLL093-3, Millipore, MA, USA). After blocking with 5% skimmed dry milk, the blots were incubated overnight with primary antibodies against: IDO1 (ab211017), caspase-3 (ab13847), cleaved-caspase-3 (ab2302), caspase-6 (ab185645), cleaved-caspase-6 (ab2326), caspase-9 (ab32539), cleaved-caspase-9 (ab2324), caspase-8 (ab108333), cleaved-caspase-8 (CST#9496), LC3 (ab48394), Beclin1 (ab210498), P62 (ab109012), and GAPDH (ab181602) at 4°C. Next, the membranes were then probed with HRP-conjugated secondary antibodies, and the positive bands were visualized using the enhanced Chemiluminescence (ECL) detection kit (GE2301-25ML, Genview, USA) on an imaging system (Bio-Rad, USA). The band densities were analyzed using Image J software, and the relative protein expression levels were normalized to that of GAPDH.

### Real-Time Quantitative PCR

Total RNA was extracted from the uLMS cells using ES-science RNA-Quick Purification Kit, cDNA synthesized with the Fast All-in-One RT Kit, and RT-PCR was performed using 2×Super SYBR Green qPCR Master Mix (PN001, YiShan Biotech, Shanghai, China) according to the manufacturer’s instructions on the ABI-7500 Sequence Detection System (Applied Biosystems, Foster City, CA, USA) with the following program: pre-incubation at 95°C for 5 min, followed by 40 cycles of denaturation at 95°C for 10 s, and then annealing and extension at 60°C for 30 s. The primers were designed as follows: IDO1 F: GCCAGCTTCGAGAAAGAGTTG; R: ATCCCAGAACTAGACGTGCAA, GAPDH F: GAGCGAGATCCCTCCAAAAT; R: GGCTGTTGTCAACTTCTCATGG. The comparative cycle threshold (Ct) method (2−ΔΔCt) was used to calculate the fold change in RNA expression. The relative mRNA levels of IDO1 were normalized to that of GAPDH. Experiments were independently performed in triplicates.

### Mouse Xenograft Tumor Model

BALB/c nude mice (6–7 weeks, female, 18 ± 2 g) were purchased from Beijing Vital River Laboratory Animal Technology (Beijing, China). Approximately 1 × 10^7^ SK-UT-1 cells were suspended and injected subcutaneously into their right flanks and once the tumor volume reached approximately 0.5 cm^3^, the mice were randomly divided into three groups (n = 5 per group) and injected intraperitoneally (i.p.) with 100 mg/kg or 200 mg/kg curdione or the same volume physiological saline (control) every day. The tumor volume (length × width^2^/2) and body weight were calculated every three days. 21 days later, the mice were euthanized, and the tumor tissues were harvested for immunohistochemical staining and western blotting. All experimental protocols were performed according to the Guidelines for the Care and Use of Laboratory Animals by the National Institute of Health, and approved by the Ethics Committee of Capital Medical University.

### Histopathology and Immunohistochemistry

The tumor, liver, and kidney tissues were harvested and embedded in 4% paraformaldehyde, dehydrated across an ethanol gradient, and cleared in xylene. The liver and kidney tissues were fixed with 4% formaldehyde, embedded with paraffin, sectioned, and stained with hematoxylin and eosin (H&E). The tumor tissue sections were heated in citrate buffer for antigen retrieval and blocked with 10% bovine serum albumin (BSA, C1052, Beyotime, Shanghai, China). After incubating overnight with primary antibodies, the sections were probed with species-specific HRP-labeling secondary antibody. The stained cells were observed by a light microscope (Leica DM60008, Kyoto, Japan) at a magnification of ×200. The positive staining was scored according to the following criteria: (1) The positive rate: 0, less than 5%; 1, 5–25%; 2, 26–50%; 3, 51–75% and 4, greater than 75%. (2) The staining intensity: 0, negative; 1, low positive; 2, positive and 3, high positive. The expression score is the multiplication of the positive rate score and staining intensity score.

### Statistical Analysis

Statistical analysis was performed using SPSS version 19.0 software (SPSS Inc., IL, USA) and GraphPad Prism 7.0 software. One-way analysis of variance ANOVA was used for comparative analysis of the significant differences among groups. Data are presented as mean ± SD of three independent experiments. *P <*0.05 was considered statistically significant.

## Results

### Curdione Decreased the Viability of uLMS Cells

**Figure 1:**
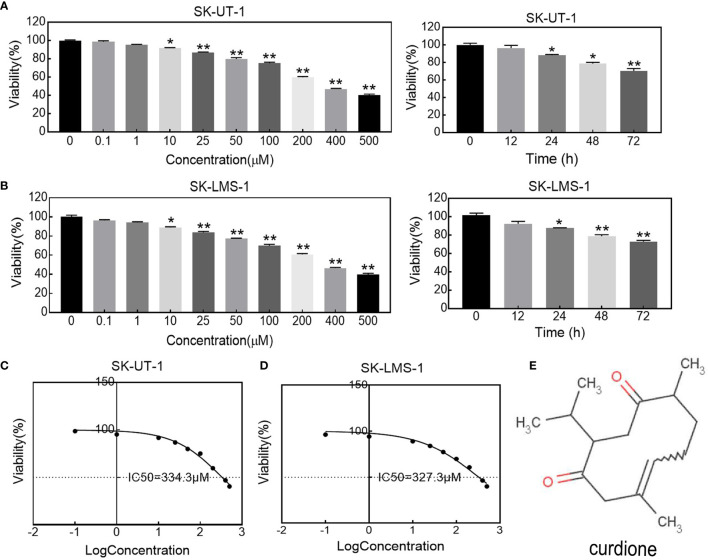
Effect of curdione on the viability in uLMS cells. The dose and time response of curdione in **(A)** SK-UT-1 and **(B)** SK-LMS-1 cells. The viability of cells treated with varying concentrations of curdione for 24 h, or 100 μM curdione for 12, 24, 48 and 72 h were detected by CCK8. Accordingly, IC50 of **(C)** SK-UT-1 and **(D)** SK-LMS-1 cells were calculated by non-linear regression using GraphPad Prism7. **(E)** Chemical structure of curdione. All values were expressed as the mean ± SD, n = 3. ^*^ *P* **<** 0.05, ^**^ *P* **<** 0.01 compared with control.

The effect of curdione on uLMS cells’ viability was detected by CCK8 assay. As shown in **Figures 1A, B**, curdione significantly decreased cell viability in a concentration and time-dependent manner at a dose higher than 10 μM. Its half-maximal inhibitory concentration (IC50) and the corresponding 95% confidence intervals for (**Figure 1C**) SK-UT-1 were 327.0 (297.7–362.8) μM and (**Figure 1D**) SK-LMS-1 cells were 334.3 (309.9–362.5) μM respectively. Accordingly, less than one-third of IC50 curdione (100 μM) were used for the subsequent experiments. The chemical structure of curdione is shown in **Figure 1E**.

### Curdione Inhibited the Proliferation of uLMS Cells

**Figure 2:**
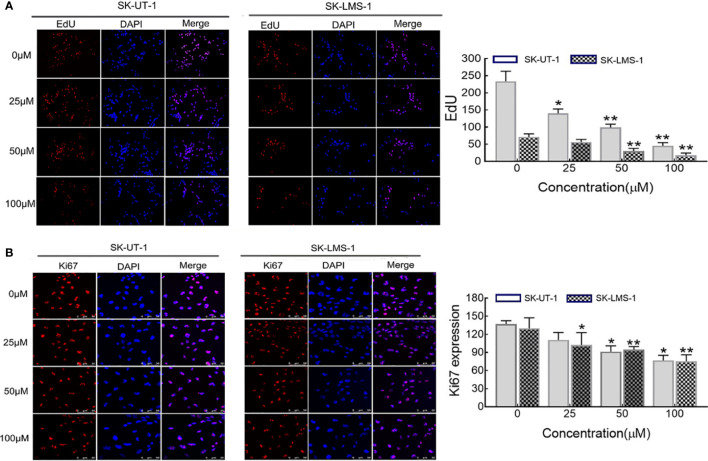
Effect of curdione on the proliferation of uLMS cells. Immune fluorescence detection of the proliferation effect of curdione in uLMS cells. Cultured SK-UT-1 and SK-LMS-1 cells were treated with 0, 25, 50, and 100 μM curdione for 24h, and stained with **(A)** EdU and **(B)** Ki67, the stained cells were observed by fluorescence microscope (×40 magnification). Quantitative analysis of the positive fluorescence density by Image J, Independent experiments were performed three times. Data were presented as mean ± SD, n = 3. *P < 0.05, **P < 0.01 compared with control.

The anti-proliferative effect of curdione on SK-UT-1 and SK-LMS-1 cells were assessed by EdU incorporation, and ki67 expression levels using immunofluorescent techniques. As shown in **Figures 2A, B**, curdione decreased the expression of both EdU and ki67 positive of SK-UT-1 and SK-LMS-1 cells in a concentration-dependent manner.

### Curdione Induced G2/M Phase Arrest in uLMS Cells

**Figure 3:**
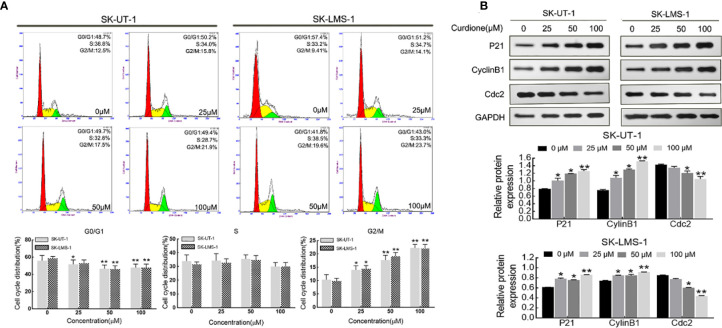
Curdione induces G2/M phase arrest in uLMS cells. **(A)** Flow cytometry analyzes the cell cycle distribution in SK-UT-1 and SK-LMS-1 cells treated with 0, 25, 50, and 100 μM curdione for 24 h. **(B)** Western blotting detection of the P21, CylinB1, and Cdc2 expression in SK-UT-1 and SK-LMS-1 cells treated with 0, 25, 50, and 100 μM curdione for 24 h. Independent experiments were performed three times. All data were presented as mean ± SD, n = 3. *P < 0.05, **P < 0.01 compared with control.

Flow cytometry analysis and Western blotting were performed on uLMS cells to explore the effect of curdione on the cell cycle. As shown in **Figure 3A**, in SK-UT-1 cells, the G2/M phase proportion of the control, 25, 50, and 100μM curdione groups were 10.34 ± 1.54%, 14.03 ± 1.28%, 17.70 ± 1.48%, and 22.27 ± 1.05%; in SK-LMS-1 cells, the G2/M phase proportion of the control, 25, 50, and 100μM curdione groups were 9.84 ± 0.83%, 14.47 ± 0.97%, 19.10 ± 1.16% and 22.27 ± 1.05%; at the same time, the G1 phase proportion of both cell lines decreased. These data revealed that, curdione markedly increased the proportion of uLMS cells in the G2/M phase and decreased that in the G1 stage, compared with control. Consistent with this, curdione also up-regulated the cell cycle checkpoint proteins P21 and CyclinB1 and down-regulated Cdc2 in a concentration-dependent manner (**Figure 3B**). These results indicated that curdione arrested uLMS cells in the G2/M phase.

### Curdione Induced Apoptosis in uLMS Cells

**Figure 4:**
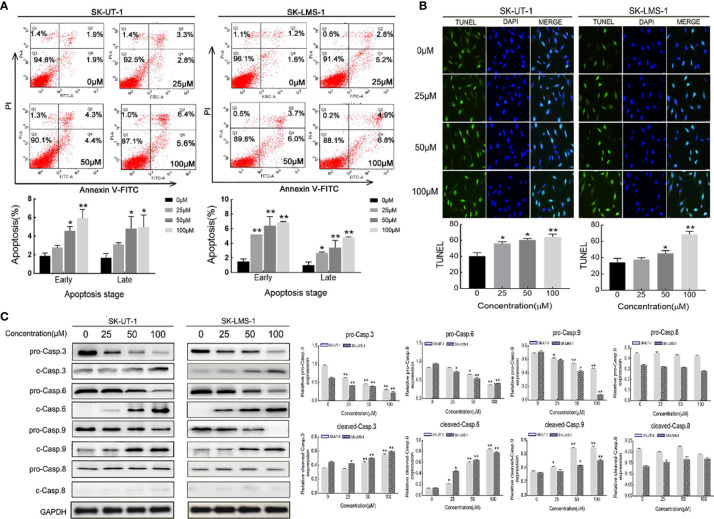
Curdione induces caspases-mediate apoptosis in uLMS cells. SK-UT-1 and SK-LMS-1 cells were treated with 0, 25, 50, and 100 μM curdione for 24 h, **(A)** Flow cytometry with Annexin V-FITC/PI staining was conducted to analyze the apoptosis ratio. **(B)** Immunofluorescent analysis with TUNEL staining was performed to detected apoptosis using laser confocal microscopy (×40 magnification), Scale bars = 100 μm. **(C)** Western blotting detection of the pro-and cleaved-caspase 3, 6, 8, and 9 expressions. The relative protein expression level was normalized to that of GAPDH. Independent experiments were repeated three times, all data were present with means ± SD, n = 3. *P < 0.05, **P < 0.01 compared with control.

The apoptosis of SK-UT-1 and SK-LMS-1 cells exposed to varying concentrations of curdione was evaluated by Annexin V-FITC/PI and TUNEL assays. As shown in **Figure 4A**, in SK-UT-1 cells, the early apoptosis rates of the control, 25, 50, and 100μM curdione groups were 1.90 ± 0.25%, 2.77 ± 0.21%, 4.57 ± 0.39%, and 5.93 ± 0.77%, the late apoptosis rates of the control, 25, 50, and 100μM curdione groups were 1.70 ± 0.36%, 3.10 ± 0.16%, 4.83 ± 1.05% and 4.97 ± 1.08%; in SK-LMS-1 cells, the early apoptosis rates in the control, 25, 50, and 100μM curdione groups were 1.50 ± 0.29%, 5.20 ± 0.01%, 6.40 ± 1.01% and 6.87 ± 0.09%, the late apoptosis rates in the control, 25, 50, and 100μM curdione groups were 1.00 ± 0.36%, 2.67 ± 0.12%, 3.40 ± 0.80% and 4.77 ± 0.09% respectively. These data indicated that curdione markedly increased the percentage of both early and late apoptotic cells in a concentration-dependent manner. Likewise, the TUNEL assay further confirmed that curdione induced apoptosis (**Figure 4B**). Furthermore, curdione increased cleaved-caspase 3, 6, and 9 in a concentration-dependent manner without affecting that of caspase 8 (**Figure 4C**). Thus, curdione induced caspases mediate apoptosis through the intrinsic pathway.

### Curdione Induced Autophagy in uLMS Cells

**Figure 5:**
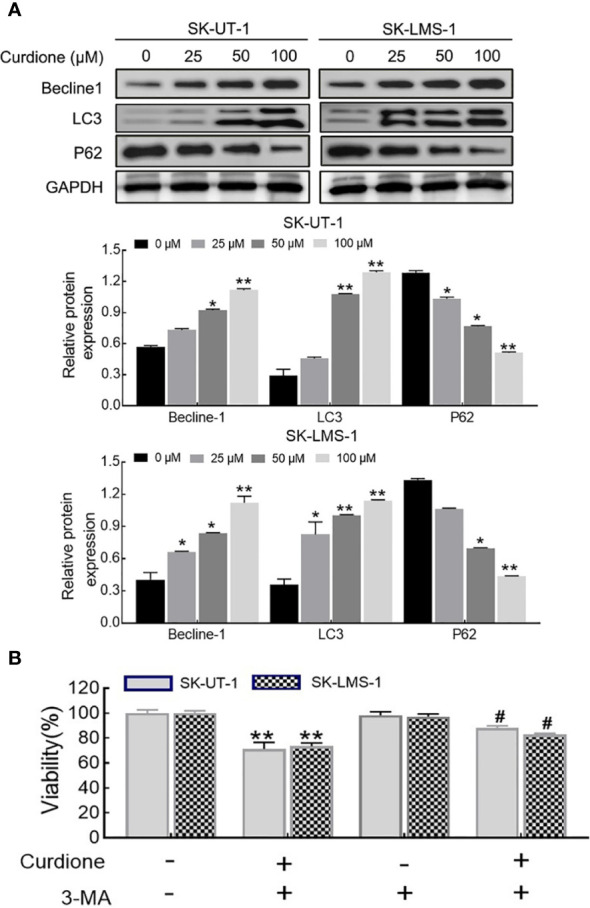
Curdione induces autophagic death in uLMS cells. **(A)** Curdione induced autophagy. Beclin1, LC3, and P62 expression in SK-UT-1 and SK-LMS-1 cells treated with 0, 25, 50, and 100 μM curdione for 24 h were determined by Western blotting. **(B)** The autophagy induced by curdione was pro-death. The viability of SK-UT-1 and SK-LMS-1 cells treated with 3-MA (2 mM) for 2 h following with curdione (100 μM) for 24 h was detected by CCK8. Statistical analysis data were present with means ± SD, n = 3. *P < 0.05, **P < 0.01 compared with control; ^#^ *P* < 0.05; ^##^ *P* < 0.01 compared with curdione alone group.

To examine whether curdione induced autophagy in SK-UT-1 and SK-LMS-1 cells, we analyzed the levels of characteristic markers including LC3, Beclin-1, and P62 by Western blotting. As shown in **Figure 5A**, curdione up-regulated LC3 and Beclin-1 and down-regulated P62 in a dose-dependent manner. To further clarify the autophagy was pro-survival or pro-death, CCK8 assay was performed in uLMS cells pre-treated with 3-MA for 2 h followed with curdione for 24 h. The viability of the curdione alone group and 3-MA pre-treated group was 71.21 ± 4.27% and 87.96 ± 1.44% in SK-UT-1 cells, 73.44 ± 2.11% and 82.94 ± 0.73% in SK-LMS-1 cells. This suggests that 3-MA partly alleviated the suppressive effect of curdione on the uLMS cells (**Figure 5B**). Taken together, the inhibitory effect of curdione on uLMS cells can also be attributed to autophagic death.

### IDO1 Mediated the Suppressive Effect of Curdione in uLMS Cells

**Figure 6:**
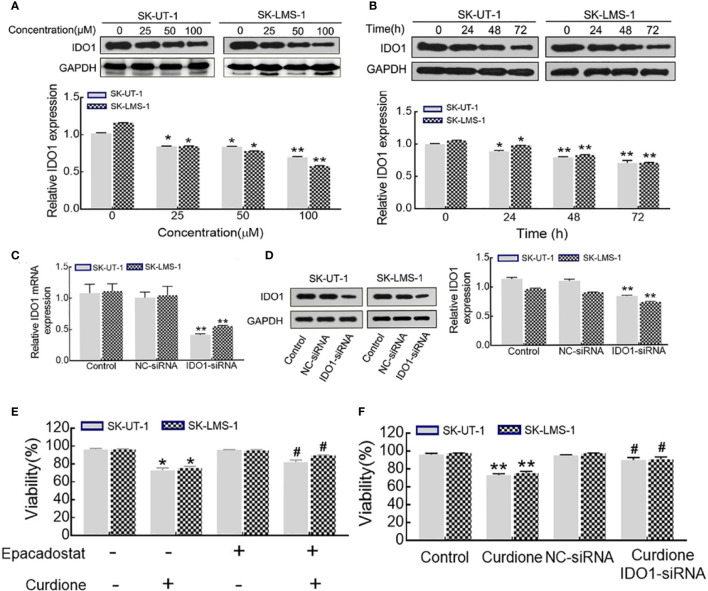
IDO1 knockdown reversed the anti-proliferation effect of curdione in uLMS cells. **(A)** Curdione down-regulated IDO1 expression in a dose-dependent manner. IDO1 expression of SK-UT-1 and SK-LMS-1 cells treated with 0, 25, 50, 100 μM curdione for 24 h were analyzed by Western blotting. **(B)** Curdione down-regulated IDO1 expression in a time-dependent manner. IDO1 expression of uLMS cells treated with 100 μM curdione for 24, 48, 72 h was analyzed by Western blotting. **(C)** mRNA levels of IDO1 in uLMs cells after transfections with IDO1-siRNA was detected by RT-PCR, **(D)** Protein levels of IDO1 in uLMs cells after transfections with IDO1-siRNA was detected by Western blotting. **(E)** Epacadostat attenuated the suppressive effect on uLMS cells. The viability of uLMS cells pre-treated with IDO1 inhibitor epacadostat (25 nM) following with curdione was detected by CCK8. **(F)** IDO1-siRNA reversed the suppressive effect on uLMS cells. The viability of uLMS cells transfected with IDO1-siRNA was detected by CCK8. All data were present with means ± SD, n = 3. *P < 0.05, **P < 0.01 compared with control; ^#^ *P* < 0.05, ^##^ *P* < 0.01 compared with curdione group.

Western blotting was performed to evaluate the effect of curdione on IDO1 expression, the results showed that, curdione significantly down-regulated IDO1 expression of uLMS cells in a dose and time-dependent manner (**Figures 6A, B** **)**. To study the effect of IDO1 on the proliferation of curdione, we transfected SK-UT-1 and SK-LMS-1 cells with siRNA to silence IDO1 expression. The stable transfection of IDO1 was confirmed by the mRNA (**Figure 6C**) and protein (**Figure 6D**) expression. In addition, the effect of IDO1 on uLMS cell viability was assessed by CCK8. The viability of the epacadostat pre-treated group and curdione alone group was 72.89 and 82.04% in SK-UT-1 cells, 75.26 and 89.41% in SK-LMS-1 cells. Similarly, IDO1-siRNA restored the viability reduced by curdione from 73.05 to 90.09% in SK-UT-1 cells, from 75.26 to 90.60% in SK-LMS-1 cells. These results suggested that, both pharmacological inhibitor epacadostat and IDO1-siRNA reversed the suppressive effect of curdione on uLMS cells (**Figures 6E, F** **)**. Briefly, the suppressive effect of curdione on uLMS cells is mediated by IDO1.

### IDO1 Mediated Apoptosis, Autophagy, and G2/M Phase Arrest Induced by Curdione in uLMS Cells

**Figure 7:**
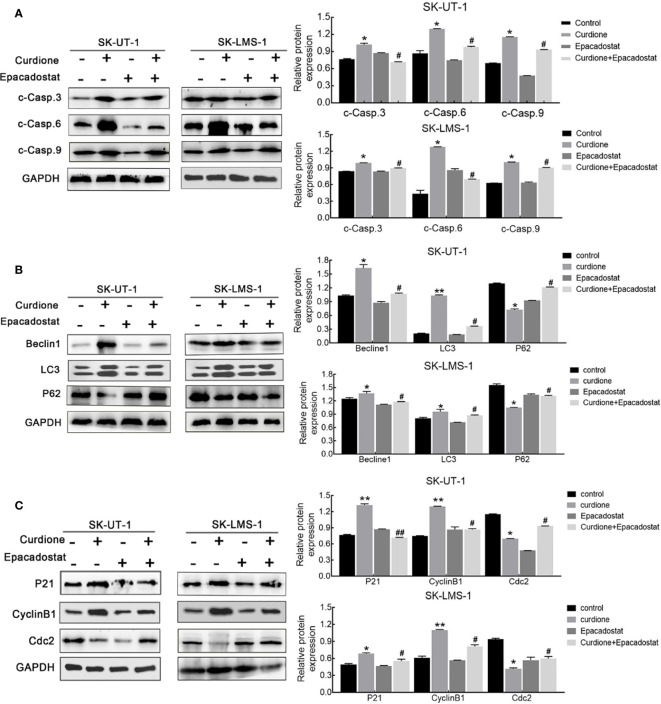
IDO1 mediates apoptosis, autophagy, and G2/M phase arrest induced by curdione in uLMS cells. Western blotting detection of **(A)** cleaved caspase 3, 6, 9; **(B)** Beclin1, LC3, and P62; **(C)** P21, CylinB1, and Cdc2 expression in SK-UT-1 and SK-LMS-1 cells pre-treated with epacadostat (25 nM) for 2 h following with curdione for 24 h. All data were presened with mean ± SD, n = 3. *P < 0.05; **P < 0.01 compared with control; ^#^ *P* < 0.05, ^##^ *P* < 0.01 compared with curdione alone group.

To clarify the mechanism underlying the ability of IDO1 to mediate the suppressive effect of curdione in uLMs cells, western blotting was performed. The result showed that, epacadostat markedly attenuated the curdione-induced changes in cleaved-caspase 3, cleaved-caspase 6, and cleaved-caspase 9 (**Figure 7A**), LC3, Beclin1, P62 (**Figure 7B**); P21, CyclinB1, and Cdc2 (**Figure 7C**), indicating that IDO1 mediates the suppressive effect of curdione in uLMs cells through regulating apoptosis, autophagy, and G2/M phase arrest.

### Curdione Suppressed the Growth of uLMS *In Vivo*

**Figure 8:**
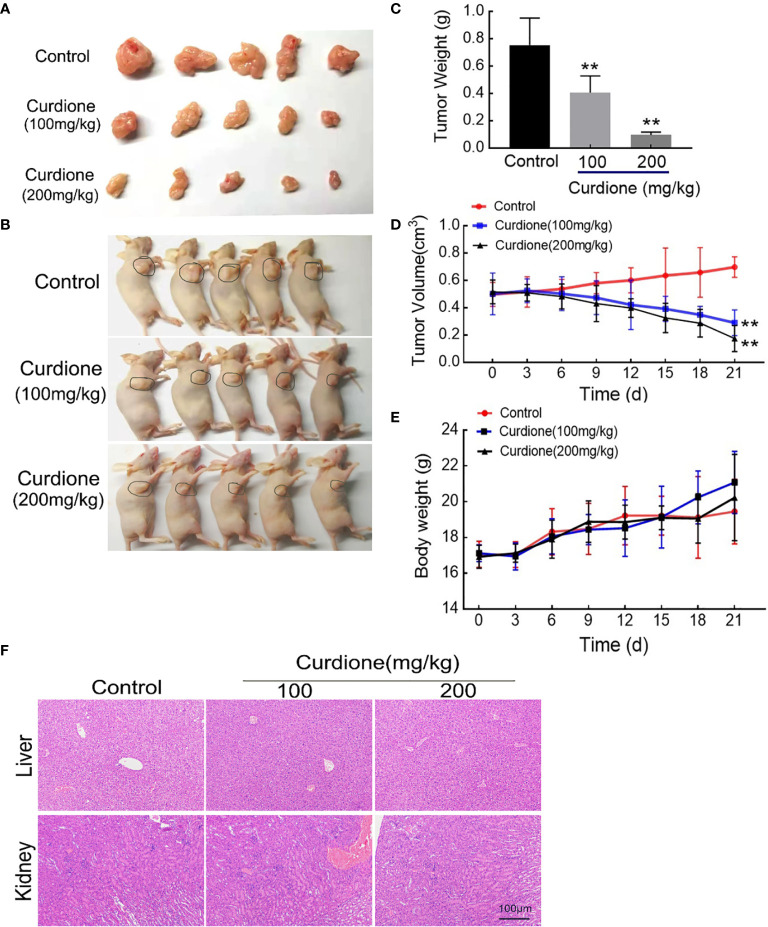
Anti-growth effect of curdione in the SK-UT-1 xenograft tumor model. SK-UT-1 xenograft tumor models were established, and randomly divided into three groups (n = 5), and then injected intraperitoneally (i.p.) with 100 or 200 mg/kg/d curdione, or same volume saline. Tumor volume and body weight were measured every three days. 21 days later, all mice were sacrificed. Representative images of **(A)** tumor-bearing nude mice and **(B)** subcutaneous dissection of tumor tissue were photographed, **(C)** tumor weight, **(D)** tumor volume, and **(E)** body weight was determined and recorded. **(F)** The pathological injury of liver and kidney tissues was assessed by the H&E staining (×200 magnification). Scale bars = 100 μm. All data were presented with mean ± SD, n = 3. *P < 0.05, **P < 0.01 compared with control.

To further explore the anti-uLMS effect of curdione *in vivo*, we established the subcutaneous xenograft models and administered them with (1) the control group: same value saline, (2) 100 mg/kg/day curdione, (3) 200 mg/kg/day curdione for 21 days. The anti-tumor effects were evaluated by tumor volume and tumor weight, and the *in vivo* safety was assessed in terms of body weight and histopathological changes. As shown in **Figures 8A–E**, after 21 days treatment, tumor weight of the control group, 100 mg/kg/day curdione and 200 mg/kg/day curdione group was (0.75 ± 0.18) g, (0.41 ± 0.11) g. and (0.10 ± 0.02) g, respectively; tumor volume of the control group, 100 mg/kg/day curdione and 200 mg/kg/day curdione group was (0.70 ± 0.07) cm^3^, (0.29 ± 0.08) cm^3^, and (0.17 ± 0.09) cm^3^. These results indicated that curdione markedly reduced tumor volume and weight without affecting body weight. Furthermore, no histopathological lesions were observed in the liver and kidney tissues (**Figure 8F**). Thus, curdione exhibited anti-uLMS growth efficacy with minimal systemic toxicity *in vivo*.

### IDO1 Mediated the Anti-Growth Effect of Curdione in the SK-UT-1 Xenograft Tumor Model

**Figure 9:**
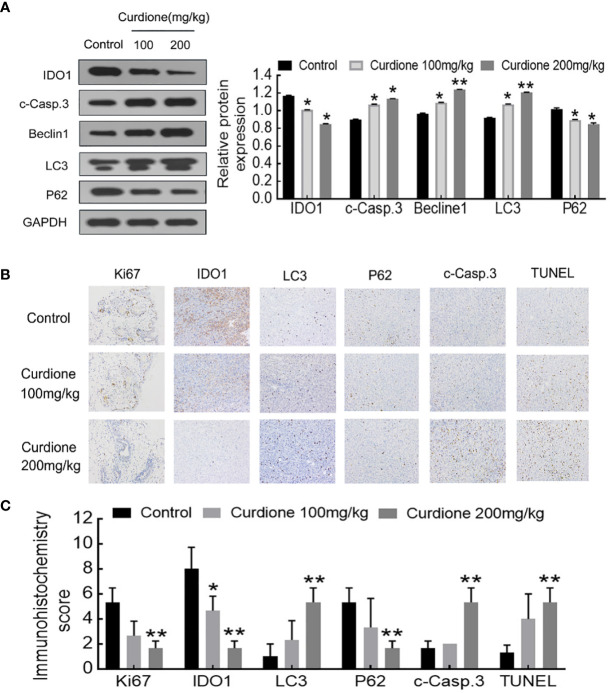
IDO1 mediates the suppressive effect of curdione in the SK-UT-1 xenograft tumor model. **(A)** Western blotting detection of protein expression of IDO1, cleaved caspase-3, Beclin1, LC3, p62 in tumor tissue. **(B)** Immunohistochemistry staining of Ki67, IDO1, LC3, p62, cleaved caspase-3, and TUNEL in tumor tissue (×200 magnification), Scale bars = 100 μm. **(C)** Expression scores of Ki67, IDO1, LC3, p62, cleaved caspase-3, and TUNEL was calculated according to immunostaining intensity and positive expression rate. All statistical analysis data were present with mean ± SD, n = 3. *P < 0.05, **P < 0.01 compared with control.

To determine the mechanism involved in the inhibitory effect of curdione on uLMS *in vivo*, western blotting and histological immunohistochemistry assay were performed. Consistent with the *in vitro* results, curdione down-regulated IDO1, ki67, and p62, and up-regulated the cleaved caspase-3, Beclin1 and LC3 in tumor tissues (**Figures 9A–C**). The above results showed that curdione suppressed the growth of uLMS *in vivo* by targeting IDO1 and activating apoptosis and autophagy.

## Discussion

Chemotherapy is a widely accepted strategy for treating cancers. However, conventional chemotherapeutic drugs have the disadvantages of high costs and adverse effects. Characteristiced by high efficacy, low toxicity, and relatively lower costs, natural plant derived compounds are favored in the field of cancer treatment, especially after the successful clinical application of paclitaxel and vinblastine (24). In fact, 80% of the chemotherapeutic drugs approved by the United States Food and Drug Administration in the past 30 years are either plant-derived compounds or their synthetic derivatives (30). *Curcuma zedoary*, a traditional Chinese medicine formulation that promotes blood circulation, removes blood stasis, and alleviates pain, has been widely used for treating gynecological diseases. Curcumin is one of the bioactive compounds of *Rhizoma Curcumae* with anti-proliferation effects on uterine sarcoma (18, 31), and uterine leiomyosarcoma (32, 33). Curdione is an active component of *Rhizoma Curcuma;* it has the same family and genus source with curcumin and structurally similar to curcumin, which leads us to hypothesize that it might have an analogous effect on uLMS.

Based on the hypothesis, we first determined the effect of curdione on cell viability. Indeed, curdione inhibited the viability of SK-UT-1 and SK-LMS-1 cells in a dose and time-dependent manner. Accordingly, the IC50 was observed, and less than a third of IC50 was used in the subsequent experiment to ensure fidelity and reliability. The anti-proliferative effect of curdione was further confirmed by the decreased EdU and Ki67-positive fluorescence images in uLMS cells. In addition, curdione contributed to the alleviation of tumor load *in vivo* with few adverse effects. These data overwhelmingly illustrated that curdione has anti-tumor effect on uLMS. Cell proliferation is regulated by cell cycle checkpoints, thus, cell cycle progression is often used to evaluate the efficacy of anti-tumor drugs. In this study, curdione increased the cell population of the G2/M phase in a concentration-dependent manner, indicating that curdione suppressed the proliferative effect of uLMS cells by inducing cell cycle arrest at G2/M.

Apoptosis and autophagy are the two common death types induced in tumor cells by chemotherapeutic drugs and various plant-derived compounds (24). Apoptosis is crucial for maintaining the dynamic balance between cell survival and death (21). It is initiated through the intrinsic (mitochondrial) pathway or extrinsic (death receptor) pathways depending on the triggering factor, and then starts caspases mediated cascade reaction, and eventually leads to cell death (22). The cleaved caspases 3, 6, and 9 mainly participate in the intrinsic pathway. Curdione enhanced the apoptosis *in vitro* and *in vivo* in a dose-dependent manner, which was associated with an increase in the levels of cleaved caspases 3, 6, and 9 but that of pro-and cleaved-caspase 8, which suggested that curdione induces caspase-mediated apoptosis through the intrinsic pathway. This is associated with Li’s (34) reported that *β*-elemene induced caspase-mediated mitochondrial cell apoptosis. Autophagy is a self-digestion process that is activated in response to various stresses and ensures cell survival by recycling proteins and organelles, which play an important role in the growth and proliferation of various sarcoma as well (35), and autophagic death is the mechanistic basis of the action of several phytochemicals. We found that curdione increased the autophagic flux in uLMS by up-regulating LC3 and Beclin-1 and degrading p62. Interestingly, the suppressive effect of curdione on the uLMS cells was completely abrogated by the autophagy inhibitor 3-MA, indicating that curdione induced autophagic cell death in uLMS.

Tumor immunotherapy has been proved to be successful in clinical application, for instance, the immune checkpoint inhibitors have greatly improved the clinical prognosis of a subset of melanoma patients (36). The immune checkpoint IDO1 is highly expressed in many tumor types and is associated with poor overall survival and worse outcome (26, 37–39), indicating its potential as a therapeutic target (40). In the current study, curdione suppressed uLMS proliferation and down-regulated IDO1 expression. To further explore the correlation between them, the viability of uLMS cells pre-treated with IDO1 siRNA or IDO1 specific inhibitor epacadostat was detected. Surprisingly, the inhibitory effect of curdione on the uLMS cells was markedly weakened by blocking IDO1 through pharmacological or genetic means, which also reversed apoptosis, autophagy, and G2/M phase arrest induced by curdione. Thus, curdione exerts its anti-neoplastic effects on uLMS through targeting IDO1.

The limitation of this study should be addressed. Acting as a “brake” or “accelerator” in tumor immune regulation, IDO1 induces immunosuppressive tumor microenvironment and enables cancer cells to escape immune surveillance by catabolizing tryptophan into tryptophan (41). However, Thaker (29) reported that IDO1 promotes colon cancer growth directly independent of T cell-mediated immune regulation. We found that IDO1 mediated the anti-proliferative effect of curdione on uLMS, further research is needed to evaluate whether the immunomodulatory effects of IDO1 play a role in inhibiting uLMS growth. Analyzing the tryptophan and kynurenine levels, as well as the immune landscape of tumors can provide more insights.

Furthermore, the focus of this study is to investigate the efficacy of curdione on uterine leiomyosarcoma and analyze its mechanism. Therefore, a series of experiments of uLMS cell lines were performed to detect the anti-tumor effect of curdione, two cell lines mutual authentication, which strongly illustrates the anti-uLMS effect of curdione. In this work, we preliminarily explore the efficiency of curdione on uLMS, it does not involve the influence on normal cells. It is indisputable that including the normal cells would be more convincing to investigate the adverse effect of curdione *in vitro*. A great majority of experiments will be needed to further verify its effectiveness and safety, in the subsequent experimental research on the toxicity and safety evaluation of curdione, including the non-cancerous cell study will be necessary.

To summarize, we have demonstrated the anti-uLMS effect of curdione *in vitro* and *in vivo*, and elucidated the underlying mechanisms. *In vitro*, curdione suppressed uLMS cell proliferation by inducing G2/M phase arrest, apoptosis, and autophagy *via* targeting IDO1. *In vivo*, it markedly reduced the tumor growth in subcutaneous xenograft tumor models by down-regulating IDO1 and activating apoptosis and autophagy. Briefly, curdione inhibited uLMS through IDO1 mediated apoptosis, autophagy, and G2/M phase arrest, it is a promising drug for treating uLMS that warrants clinical investigation.

## Data Availability Statement

The original contributions presented in the study are included in the article/supplementary material. Further inquiries can be directed to the corresponding author.

## Ethics Statement

The animal study was reviewed and approved by the Ethics Committee of Capital Medical University.

## Author Contributions

CW and DL conceived and designed the experiments. CW performed the experiments and wrote the manuscript. YL, WW, and TQ contributed to the experimental study design, preparation, and review of this manuscript. DL was responsible for manuscript revision and financial support. All authors contributed to the article and approved the submitted version.

## Funding

This work was supported by the National Natural Science Foundation of China (no. 81774072, 81373812, 81073096), the Natural Science Foundation of Beijing Municipality (no. 7202015).

## Conflict of Interest

The authors declare that the research was conducted in the absence of any commercial or financial relationships that could be construed as a potential conflict of interest.
